# The Coronavirus Nucleocapsid Is a Multifunctional Protein

**DOI:** 10.3390/v6082991

**Published:** 2014-08-07

**Authors:** Ruth McBride, Marjorie van Zyl, Burtram C. Fielding

**Affiliations:** Molecular Biology and Virology Research Laboratory, Department of Medical Biosciences, Faculty of Natural Sciences, University of the Western Cape, Private Bag X17, Modderdam Road, Bellville, Western Cape 7535, South Africa; E-Mails: rmcbride@uwc.ac.za (R.M.); 2917799@myuwc.ac.za (M.Z.)

**Keywords:** nucleocapsid protein, coronavirus assembly, coronavirus N, intracellular localization, protein topology

## Abstract

The coronavirus nucleocapsid (N) is a structural protein that forms complexes with genomic RNA, interacts with the viral membrane protein during virion assembly and plays a critical role in enhancing the efficiency of virus transcription and assembly. Recent studies have confirmed that N is a multifunctional protein. The aim of this review is to highlight the properties and functions of the N protein, with specific reference to (i) the topology; (ii) the intracellular localization and (iii) the functions of the protein.

## 1. Introduction

Coronaviruses (CoVs) have a global distribution and infect a variety of human and animal hosts, causing illnesses that range from mostly upper respiratory tract infections in humans to gastrointestinal tract infections, encephalitis and demyelination in animals; and can be fatal [1,2]. The International Committee for Taxonomy of Viruses (ICTV) reports four coronavirus genera, namely *Alphacoronaviruses*, *Betacoronaviruses*, *Gammacoronaviruses* and *Deltacoronaviruses* [3]. CoVs are enveloped single-stranded, positive-sense RNA viruses with genomes ranging between 26.2–31.7 kb, the largest among known RNA viruses [4]. This large, capped and polyadenylated genome contains seven common coronavirus genes in the following conserved order: 5'-ORF1a-ORF1b-S-ORF3-E-M-N-3' [5]. ORF1a/b encompasses two-thirds of the genome and produces a genome-length mRNA (mRNA1) that encodes two overlapping viral replicase proteins in the form of polyproteins 1a (pp1a) and pp1ab [6].

These polyproteins are formed as a result of a -1 ribosomal frame shift that involves a complex pseudoknott RNA structure [7] and are then proteolytically processed by virally encoded proteases into mature nonstructural proteins (nsp1 to nsp16), which assemble to form a membrane-associated viral replicase-transcriptase complex (RTC) [6,8,9]. The last third of the genome produces subgenomic (sg) mRNAs that encode the four structural proteins, spike (S), envelope (E), membrane (M), and nucleocapsid (N), as well as a number of accessory proteins [10,11]. 

## 2. Topology of CoV N and RNA Binding

**Figure 1:**
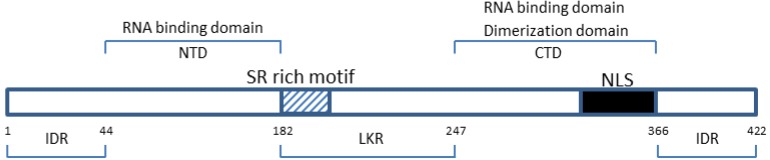
Domain organization of the Severe Acute Respiratory Syndrome human coronavirus (SARS-CoV) nucleocapsid protein. IDR (a.a. 1–44; 182–247; 366–422)—intrinsically disordered regions; NTD (a.a. 45–181)—N terminal domain; LKR (182–247)—linker region; CTD (248–365)—C-terminal domain. The charged SR rich (striated box) and the nuclear localization signal (NLS, solid box) motifs are shown [16,18,19].

Amino acid sequence comparisons have shown that CoV N proteins have three distinct and highly conserved domains: two structural and independently folded structural regions, namely the N terminal domain (NTD/domain 1) and C-terminal domain (CTD/domain 3), which are separated by a intrinsically disordered central region (RNA-binding domain/domain 2) (Figure 1); all three domains have been shown in different CoVs to bind with viral RNA [12,13,14,15,16,17].

The NTD is divergent in both sequence and length. It has been mapped for Infectious Bronchitis Virus (IBV)-N to aa 19–162 [20], for Severe Acute Respiratory Syndrome human coronavirus (SARS)-N to aa 45–181 [16], and for Mouse hepatitis Virus (MHV)-N to aa 60–197 [18]. The N-termini of these three CoVs have been found to associate with the 3' end of the viral RNA genome, possibly through electrostatic interactions [21,22]. There are several common characteristics of CoV N protein NTDs, including predicted secondary structures such as a central β-sheet platform flanked by α-helices [20], with a basic RNA binding groove along the β-platform and an extended β-hairpin. The NTD is enriched in aromatic and basic residues and the folded shape resembles a hand with basic fingers that extend far beyond the protein core, a hydrophobic palm, and an acidic “wrist” [21]. It has been proposed that the flexible, positively charged finger-like β-hairpin extension in the NTD of both IBV and SARS-CoV N protein is able to grasp RNA by neutralizing its phosphate groups, while the base moieties can make contact with exposed aromatic residues from the hydrophobic palm [16,21]. More precise mapping of the RNA-binding site locations has been determined for SARS- and IBV-N protein. Within the NTD of SARS-CoV-N, positively charged lysine and arginine residues have been proposed to bind a 32 nucleotide stem-loop structure located at the 3' end of the SARS-CoV RNA genome [16]. Site-directed mutagenesis studies on IBV-N have identified two residues that are critical for RNA binding; namely Tyr-94 and Arg-76 [23]. Tyr-94 is located in strand β3 of the four-stranded anti-parallel β sheet; Arg-76 is located in the immediate vicinity of Tyr-94, at the base of the extended flexible hairpin loop [23]. It is however likely that, since no single mutation totally disrupts RNA binding, other aromatic/basic residues at the surface of the NTD contribute to nucleic acid binding by creating a broad surface that comes into contact with the viral genomic RNA [23]. The NTD possesses some features similar to those of other RNA-binding proteins that form a RNP. For example, the U1A spliceosomal protein [24] and the coat protein of MS2 bacteriophage [25] bind viral RNA with residues arising from the surface of a four-stranded anti-parallel β sheet. Seemingly, strands β2, β3, and the flexible β-hairpin from the IBV N protein could fulfill a comparable role by interacting with phosphate groups on the viral RNA [23]. The Arg-76 and Tyr-94 residues in the IBV N protein are well conserved across the whole CoV family, and may structurally correspond to the Arg-94 and Tyr-122 residues in the SARS-CoV N protein [23], meaning that Arg-94 and Tyr-122 may therefore be critical for SARS N-RNA binding. 

The crystal structure of MHV N197 (residues 60–197) adopts a U-shaped β-platform containing five short β-strands (arranged β4-β2-β3-β1-β5) across the platform with an extended β2'-β3' hairpin similar to NTDs from other CoV N proteins [26]. Interestingly, the crystal structure of the MHV NTD shares a similar overall and topology structure with that of SARS-CoV and IBV but varies in its potential surface, indicating a possible difference in RNA-binding module [27]. It has been shown that N219, an MHV-A59 N domain protein fragment that contains the folded NTD and the immediately adjacent intact linker region (LKR; residues 60–219), binds to the TRS in the viral genome body (TRS-B) and complementary TRS (cTRS) with high affinity to form a N219-TRS duplex [26]. MHV TRS binds across the β-platform of NTD in a defined orientation, with the 5'-end of TRS near β4 and the 3'-end of TRS near β5; this N219 binding to single-stranded RNAs—containing the TRS or cTRS—uses base stacking interactions between aromatic side chains on the β-platform with a triple adenosine motif within the TRS, 5'-gAAU**CUAAAC**U-3' [26]. Furthermore, due to its potent helix-destabilizing activity, N219 is able to efficiently melt an RNA duplex between the template TRS and nascent cTRS strand into component single strands that may be transiently formed during discontinuous transcription of viral sgRNA by the coronaviral replicase complex [26]. Three residues on the β-platform have been shown to play key roles in TRS binding and helix destabilization: Arg-125 and Tyr-127 on the β3 strand and Tyr-190 on the β5 strand, suggesting that the AAA motif in the 3'-end of the TRS is anchored here [18]. These three residues are completely invariant in betacoronavirus N proteins and occupy precisely analogous positions on the fold of each NTD, and are therefore likely to define similar RNA binding grooves in the closely related SARS NTD [18]. The duplex formation and duplex TRS unwinding activity exhibited by N219 therefore implicates MHV NTD in template switching during discontinuous sgRNA transcription [28,29]. Moreover, the ability of the NTD to melt dsRNA may also play a role in RNA packaging or other steps in the viral life cycle where RNA remodeling is needed [26]. For example, mutations that cripple duplex unwinding are defective in stimulating CoV replication in BHK-R cells, and are lethal, providing evidence of a critical role for NTD in viral replication [18,26]. CoV N proteins have also been recognized as RNA chaperones [30,31], which, as part of their chaperone activities, anneal nucleic acids, and so RNA duplex destabilization activity may be important in CoV N NTDs role in assisting viral RNA in reaching its functional three-dimentional structure. Viral nucleocapsid and replication accessory proteins from other viruses have also been shown to function as RNA chaperones and facilitate helix destabilization, for example HIV-1 NCp7 protein [32], and adenovirus DNA binding protein [33].

The NTD is separated from the CTD by an intrinsically disordered middle region referred to as the linker region (LKR). The charged LKR is also known as the SR-domain because it is rich in serine and arginine residues [34], and it is involved in cell signaling [15,35,36]. The flexible LKR is capable of direct interaction with RNA under *in vitro* conditions [37]. Potential phosphorylation sites have been mapped to the Ser/Arg-rich portion of the LKR of SARS-CoV N [38,39,40]. These LKR phosphorylation sites are thought to function in binding M protein, heteronuclear ribonucleoprotein (hnRNP-A1) and RNA to the N protein with high binding affinity [14,41,42,43]. There are conflicting reports regarding the involvement of the LKR in N protein oligomerization. Some studies has suggested that the LKR is directly involved [44] and that through electrostatic effects, hyperphosphorylation of the LKR reduces the total positive charge on the SARS-CoV N protein and leads to enhanced oligomerization of di-domain constructs [45]. Other studies have, however, reported that the LKR interferes with oligomerization when the CTD is present [46] or if the LKR is phosphorylated [38]. Despite almost no structural information being available for the LKR, possibly due to its high positive charge and flexible nature [47], there is evidence in support of the functional importance of intrinsically disordered regions in proteins for modulating transcription, translation, post-translational modifications such as phosphorylation, and cell signaling [48]. RNA chaperones often have structural flexibility because the RNA-protein recognition process often requires conformational changes in the RNA, the protein or both [49]. An interaction between N protein and a subunit of the viral replicase-transcriptase complex, namely non-structural protein 3 (nsp3), has been described and key binding determinants localize to the LKR [50,51], highlighting the importance of this unstructured region for a number of potential interactions, such as viral infectivity [52]. It has also been proposed that nsp3 binding induces a conformational change in the LKR, potentially regulating the intracellular localization of N to the site of replication [50] and/or other RNA binding functions of N. 

The CTD, which is a hydrophobic, helix-rich terminal, has been mapped for SARS-N to aa 248–365 [17], and for IBV-N to aa 219–349 [21,53]. This domain is also referred to as the dimerization domain because it contains residues responsible for self-association to form homodimers, as well as homo-oligomers through a domain-swapping mechanism [16,17,42,53,54,55,56,57]. Oligomerization of N protein is necessary to produce a stable conformation because in its monomeric form, the CTD folds into an extended conformation with a large cavity in its center, making it unstable [47]. Sequence comparison shows that the dimerization domain of the N protein is conserved at least among the alpha, beta and gamma groups of CoVs, suggesting a common structural and functional role for this domain [47]. The monomer of cSARS-N, a crystalized C-terminal construct of SARS-N that contains residues 270–370, comprises five short α-helices, one 3_10_ helix, and two β-strands [47]. The general shape of the monomer resembles the letter C, with one edge formed by a β-hairpin extending away from the rest of the molecule [47]. This structure is similar to the crystalline structure of another SARS N CTD monomer (NP248–365), which consists of eight α-helices and two β-strands [55]. The cSARS-N dimer interface is formed largely by insertion of the β-hairpin of one subunit into the cavity of the opposite subunit, resulting in the four β-strands of the two subunits forming an anti-parallel β-sheet that is superposed by two long alpha helices [47]. Due to the extensive hydrogen bond formation between the two hairpins, together with hydrophobic interactions between the beta-sheet and the alpha helices, this interface is highly stable [17], and these interactions suggests that the dimeric structure may in fact represent the functional unit of the N protein [47]. The crystal structure of NP248–365, a SARS-CoV CTD spanning residues 248–365, revealed that the N protein dimer has the shape of a rectangular slab in which the four-stranded β-sheet forms one face of the slab and the α-helices form the opposite face [55]. Similarly to cSARS N, the dimerization interface of NP248–365 is composed of four β-strands and six α-helices, with each protomer contributing one β-hairpin and helices α5, α6 and α7. The two β-hairpins form a four-stranded intermolecular β-sheet that is stabilized through extensive hydrogen bonding. The other part of the dimerization interface is composed of helices α5 and α6, where strong hydrophobic interactions involving Trp302, Ile305, Pro310, Phe315 and Phe316 were observed. The dimer is further stabilized by hydrophobic interactions between the longest helix, α7, and the intermolecular β-sheet [55]. Similarly to cSARS-N and NP248–365, a nuclear magnetic resonance (NMR) study has reported secondary structural assignments of a SARS N protein construct whose dimeric interface also consists of a four-stranded anti-parallel β-sheet and two α-helices [17]. 

Self-association of the N protein has been observed in many viruses, and is required for the formation of the viral capsid which protects the viral genome from extracellular agents [56]. In addition to the ability of N protein to oligomerize, viral capsid formation also requires RNA-binding ability [58]. Studies revealed that SARS-CoV N protein fragments containing the dimerization domain (residues 236-384) can bind to a putative packing signal within the viral RNA, with the most likely RNA binding site being within the basic region between residues 248–280 [59]. NMR studies then showed that the RNA-binding site between residues 248–280 formed part of the complete dimerization domain structure [17]. It was not until the crystal structure of SARS N CTD was resolved that the molecular basis of RNA-binding activity and organization of the CTD octamer was determined. The CTD spanning residues 248–365 (NP248–365) revealed that, due to the presence of the eight positively charged lysine and arginine residues, amino acids 248–280 form a positively charged groove, one of the most positively charged regions of the N protein [55]. This groove is similar to that in IBV-N CTD, except that the positively charged surface area is larger in the SARS-CoV construct than in the IBV [20], due in part to the presence of additional negatively charged residues in the IBV N protein and in part due to the absence of residues 215–218 from the IBV construct, which contain two lysine residue in the SARS-CoV construct [55]. The NP248–365 construct, which contains both the charge-rich region (residues 248–280) and dimerization core (residues 281–365) of the dimerization domain, is capable of binding to single-stranded RNA (ssRNA), single-stranded DNA (ssDNA) and double-stranded DNA (dsDNA), and NP248–365 has stronger nucleic acid-binding activity than the NTD [55,57]. The strong electrostatic character of residues 248–280 and the fact that both ssRNA, ssDNA and ddDNA bind to NP248–365, strongly indicates that oligonucleotide binding is based on non-specific charge interactions between the positively charged protein and the negatively charged nucleic acid backbone [55,60]. By keeping the RNA-binding domains in close proximity to the CTD, the formation of a large helical nucleocapsid core is therefore possible [47]. Association of the N protein dimers is necessary for further assembly of the core. The full-length dimeric N protein has a tendancy to form tetramers and higher molecular weight oligomers *in vitro* [54], and a serine/arginine-rich motif (residues 184–196) has been shown to be important for N protein oligomerization [44]. Two dimers arrange themselves into a butterfly-shaped tetramer, while two butterfly-shaped tetramers unite to form an octamer in the asymmetric unit of the CTD crystal [55]. The octamer is held together through hydrophobic interactions and hydrophilic contacts among the four dimers, and networks of inter-dimer hydrogen bonds further help stabilize the octamer [55]. Crystallography studies have demonstrated that CoV-SARS CTD (NP248–365) packs as an octamer which stacks to form a helical supercomplex structure with a continuous positively charged surface that could potentially allow viral RNA strands to bind and wrap around the helical oligomer structure through electrostatic interactions [55]. The existence of transient self-association between dimers in solution was confirmed using a disulphide trapping technique, and it was shown that by neutralization of excessive charges on the protein, either through environmental charge screening or charge modifications, this transient self-association can be regulated [44]. This proposed biophysical mechanism whereby electrostatic repulsion between N protein molecules acts as an oligomerization switch has implications for understanding how nucleocapsid assembly is then subsequently modulated [44]. In addition, the CTD 45 residues of the MHV N protein have been shown to be the major determinant for interaction with the M protein [61], and so association of the N protein with the M protein may also play a role in the assembly of the nucleocapsid core into a progeny virion [47].

Oligomerization via the CTD has also been reported in human coronavirus 229E (hCoV-229E) and a recent study has shown that the C-terminal tail peptide, an intrinsically disordered domain that flanks the CTD, plays an important role in dimer–dimer association [53]. The C-terminal tail interferes with oligomerization of the CTD and has an inhibitory effect on viral titer of HCoV-229E; and further understanding this mechanism of oligomerization may provide insight into the viral assembly process and could identify additional targets for drugs to combat CoVs through the disruption of the N protein self-association [53]. The CTD of SARS-N (aa 251–422) is also responsible for stress granule localization that occurs as part of an integrated stress response in arsenite-treated HeLa cells [38]. Once sequestered in these granules, the N protein can induce host translational shutoff.

The NTD and the CTD are interspersed by intrinsically disordered regions (IDRs) [19,37]. Intrinsically disordered proteins (IDPs) or IDRs lack a tertiary structure and have no fixed 3-dimentional shape in the native form. However, IDPs and IDRs play a role in various biological functions including DNA, RNA and protein binding with the disordered regions facilitating access to these binding sites [62,63,64]. 

In fact, the three IDRs in SARS-N (aa 1–44, 182–247 and 366–422) have all been shown to modulate the RND-binding activity of the NTD and CTD [19,37]. Moreover, both the middle and C-terminal IDRs (Figure 1) have been implicated in the oligomerization of the N protein [44,65], with the middle IDR also associated with N protein functionality and N-M interaction [19,39,40,66]. It would be interesting to determine whether the presence of three disordered regions in SARS N, compared to the one disordered region in HCoV-NL63 N for example, would result in SARS N having a higher binding affinity to viral, as well as host cellular proteins. If indeed so, could this then indicate a probable basis for the increased pathogenicity of SARS-CoV compared to HCoV-NL63? 

In order for CoV N proteins to package the viral genome with structural proteins to form ribonucleoprotein (RNP) complexes for viral assembly, two key activities are required: the interaction between protein and nucleic acid and the ability of the complex to oligomerize [58]. The N proteins of SARS-CoV, IBV and MHV have all been shown to perform both these functions. SARS-CoV-N protein interacts with RNA at multiple sites, with all three domains having charged regions [55]. The crystal structures of the NTD and CTD domains of the N protein from SARS-CoV, IBV and MHV all share a similar overall and topology structure, which corroborates a conserved mechanism of nucleocapsid formation for CoVs [27].

Furthermore, despite a lack of significant sequence similarity, the cSARS-N had a similar fold to that of the N protein of porcine reproductive and respiratory syndrome virus, a member of *Arteriviridae* family, suggesting an evolutionary link between *Coronaviridae* and *Arteriviridae* in which the N proteins of both viruses have a common origin [47]. In fact, due to their similar genome organization and viral replication mechanisms, the *Coronaviridae* and *Arteriviridae* were united to form the relatively new order *Nidovirales*.

## 3. Intracellular Localization of the Nucleocapsid Protein

In virus-infected cells, CoV N proteins can localize to the cytoplasm alone or to the cytoplasm and nucleolus [67]. Proteins that are able to localize to the cytoplasm, nucleus and/or nucleolus require multiple signals to determine their subcellular localization [68]. CoV N proteins commonly localize in the nucleolus, and although nucleolar localization/retention signals (NoRSs) and pathways are not well characterized, nucleolar localization usually requires regions in the protein that are rich in Arg residues and is likely cell-cycle dependent [34,69,70]. 

The N protein of IBV was found to localize in the cytoplasm alone or to co-localize in both the cytoplasm and nucleolus [67,68,70]. IBV N protein contains a functional nuclear export signal (NES) to traffic N protein to the cytoplasm [68,71], and an 8 amino acid NoRS motif at its NTD and is necessary and sufficient for nucleolar retention [68]. It is hypothesized that the localization of IBV-N to the nucleolus forms part of a virus strategy to control sgRNA synthesis in both the host cell and virus by associating with ribosomal subunits [70] and interacting with nucleolar proteins, nucleolin and fibrillarin [72]. Importantly, this interaction is not direct, but mediated through RNA and could therefore simply be an artifact of the proteins having RNA-binding domains [73]. Thus, the nucleolar localization could simply be due to a high density of the host RNA attracting a viral RNA-binding protein. Even so, it has been postulated that the nuclear localization of the N protein may interfere with cellular machinery and thus lead to triggering of apoptosis [39]. The localization of N to nucleoli alone might be cell cycle dependent, because the number and size of nucleoli differ at different stages of the cell cycle: at the beginning of G1 phase, multiple nucleoli can be found, but only single nucleoli can be seen at later G1, S and G2 phases [67,74]. It was also found that domain 2 of IBV-N predominantly localizes in the nucleus, but when fused with domain 3 (CTD) it localizes to the cytoplasm and thus supports the findings of other studies done on IBV-N localization [35,68].

The ability for nucleolar localization varies between N proteins of different CoVs. Unlike other CoV N proteins, SARS-CoV N protein is mostly distributed to the cytoplasm [34,71,75]. This cytoplasmic localization is somewhat unexpected because there is at least one NoRS in domain 2 and eight putative nuclear localization signal (NLS) motifs within domains I and II of the SARS-CoV N protein [35], of which the short lysine-rich sequence (366–381) near the carboxy-terminus is a putative bipartite NLS that is unique to SARS-CoV N [71,76]. As a reason for this, it has been suggested that signals for nuclear and nucleolar targeting of SARS-CoV N protein are poorly accessible to nuclear import machinery due to phosphorylation or conformation restraints [71]. A cytoplasmic NES may be involved in also over-riding the NLS, resulting in significantly less N protein (only 10%) being localized to the nucleolus [35]. Shuttling of N protein from the nucleus to the cytoplasm occurs through phosphorylated-dependent binding of SARS-CoV to 14-3-3, with the absence/inhibition of this 14-3-3 molecule resulting in increased nuclear localization of SARS-N [38]. Also, the deletion of the SR-rich domain contained within the middle region of SARS-N can result in dramatic changes in sub-cellular localization of N compared to wild-type N [44]. These results indicate that the localization of N protein to the nucleus or nucleolus is not a conserved property of *Nidovirales* [71].

## 4. Functions of the Nucleocapsid

### 4.1. Virus Life Cycle

#### 4.1.1. CoV N and Viral Core Formation

The primary role of CoV N protein is to package the genomic viral genome into long, flexible, helical ribonucleoprotein (RNP) complexes called nucleocapsids or capsids (Table 1). The nucleocapsid protects the genome and ensures its timely replication and reliable transmission. The filamentous nucleocapsids are 10 to 15 nm in diameter and several 100 nm in length, and these macromolecular structures are visible using electron microscopy [77]. Within the nucleocapsid there are both N-RNA interactions as well as intermolecular association between disulfide-linked N protein multimers [78]. The N-RNA interaction is mediated by binding signals contained within the leader RNA sequences [79]. During the virus life cycle, multiple copies of the N protein interact with gRNA and sgRNA molecules, indicating a role for N protein in viral transcription and translation [79,80]. The basic building block for CoV nucleocapsid formation is a dimeric assembly of N protein [21], and it is the CTD of N protein that possesses dimerization function [56].

A structural model of CoV proposes that N protein is not only present in the helical nucleocapsid but also in the internal spherical/icosahedral core [81]. The internal core consists of N protein, RNA and the CTD of M protein. The M protein is the main core shell component and a 16 amino acid domain (aa 237–252) on the CTD of M protein binds directly to N protein via an ionic interaction, leading to specific genome encapsidation in the budding viral particle [81,82,83].The N protein therefore plays an essential structural role in the CoV virion through a network of interactions with (i) the genomic RNA; (ii) M protein and (iii) other N proteins.

#### 4.1.2. CoV N and Viral Assembly

Assembly of virus particles is an essential step for a productive viral replication cycle. CoV virions contain three envelope proteins, M, E and S, and a viral nucleocapsid, which consists of genomic RNA and N protein, within the viral envelope. Assembly of CoV virions not only requires CoV N protein dimerization [44,54,56] and association with viral genomic RNA to form RNPs [43,79,81,84,85,86] but also protein-protein interactions amongst the four structural proteins, as well as a host membrane envelope obtained from the site of budding. CoVs acquire their lipid envelope by budding of the nucleocapsid through the endoplasmic reticulum (ER)-Golgi intermediate compartment (ERGIC) membranes [87,88]. It is believed that the interaction of the nucleocapsid with envelope proteins drives the incorporation of the nucleocapsid in enveloped viruses [89], and such protein-protein interactions are critical for viral assembly, as has been shown for alphaviruses [90,91]. 

N and M proteins are the two major structural proteins in CoV virions [92]. The M protein is anchored by its three transmembrane domains to the viral envelope and its large carboxy-terminal tail in the virion interior interacts with the nucleocapsid [93]. The nucleocapsid consists of the positive strand genomic RNA, mRNA 1, helically encapsidated by N protein monomers, and the N protein region that interacts with the C-terminus of the M protein domain seems to be CoV specific. The intracellular sites of virus assembly also vary among different viruses [94,95].

In MHV, the large carboxy-terminal domain of the M protein interacts with the CTD of the N protein [93]. Newly synthesized, unglycosylated M protein interacts with N protein at the ER membrane, which is a pre-Golgi compartment that is also the site of MHV budding [96], suggesting that the site of interaction overlays with MHV budding sites [93]. The M protein-nucleocapsid interaction is thought to be initiated by a direct binding of M protein to genomic RNA that is mediated by a 69 nucleotide (nt) packaging signal (ps) present only on the mRNA 1 association [93,97]. These ps is located about 21kb from the 5' end of MHV mRNA 1 [97,98], is necessary and sufficient for packaging RNA into MHV particles [99], and it has been suggested that the M protein-ps interaction could lead to the association of M protein with N protein, thereby stabilizing the complex between M protein and the nucleocapsid [93]. Although the M protein-nucleocapsid interaction could theoretically also be initiated by direct binding of N protein to genomic RNA, this is unlikely because N protein interacts with all mRNAs [79,93], which makes it difficult to explain how the formation of N protein-mRNA 1 RNP complex might lead to specific packaging of genomic RNA, and not sgRNA, into virus particles [100]. It has since been conclusively demonstrated that M protein selectively interacts with ps-containing RNA in the absence of N protein, indicating that the mechanism of M-ps recognition does not require the formation of RNP complex by N protein, and in fact, N protein is not required for RNA packaging in that model [100]. MHV M protein was the first example of a viral transmembrane protein that could bind to a specific viral RNA element in the absence of any other viral structural proteins [100], and a proposed model for RNA packaging in MHV suggests that once M protein accumulates and oligomerizes in the intermediate compartment between the ER and Golgi complex, M protein binds to ps-mRNA 1, and only thereafter does N protein associated with mRNA 1 interact with oligomerized M protein [100]. Although N protein appears to be dispensable for MHV RNA packaging, N-M interaction might be important in compensating for viral envelope defects that occur due to M protein mutation. The M protein carboxy terminus is extremely sensitive to mutations, and removal of even only the last two amino acid residues from the tail of the M protein appears to be lethal [83]. Interestingly, N protein becomes mutated in its CTD, and these changes then compensate for the loss of the two M protein residues, either by increasing the affinity of an adjacent interaction or by providing a new contact point between N and M to stabilize the virion [83].

For the porcine transmissible gastroenteritis coronavirus (TGEV), an interaction between the carboxy terminus of M and nucleocapsid has been mapped to residues 233–257 of the TGEV M protein [82]. This segment corresponds to residues 201–224 of the MHV M protein [83], which overlaps with only one of the critical residues identified in the MHV M protein [61]. This region of the two M proteins is, however, poorly conserved and the apparent disagreement between the TGEV and MHV results may relate to differences in the respective folds of the M proteins, or differences in how these residues influence those folds [61]. 

SARS-CoV is markedly different from other members of the *Coronaviridae* family in the sense that there is only 20%–30% amino acid identity with other known CoVs, with both the N and M proteins having low sequence homology [76,101]. One might therefore expect that there could be differences in the mechanism of viral assembly. A mammalian two-hybrid system, which is performed *in vivo* so that viral proteins will adopt their native state and therefore be more likely to interact in a biologically accurate manner [102], confirmed that N-M protein interactions occur *in vivo* [66]. Moreover, this study identified a stretch of amino acids (168–208) in the middle of the N gene that may be critical for N-M protein interaction [66]. This stretch of amino acids spans the LKR and dimerization domain in the CTD, suggesting that this region may be essential in maintaining correct N protein conformation for both self-association and N-M protein interaction [66]. Despite SARS-CoV having the closest genetic resemblance to MHV, the M proteins of these viruses bind to different domains on the N protein.

#### 4.1.3. CoV N and Virus Budding/Envelope Formation

CoVs assemble and bud intracellularly at the ER-Golgi complex [96,103], and association of the nucleocapsid with this organelle may reflect a role in virus budding. The formation of the virion envelope requires expression of only M- and E-protein, and not N protein, as has been observed for MHV [104], IBV [105], TGEV [106], and BCoV [107]. It was recently noted however, that these studies all used vaccinia-based expression systems, where overexpression of viral membrane proteins may lead to release in microvesicles, complicating the interpretation of virus-like particle (VLP) experiments [108]. Subsequent experiments using transient transfection to express the proteins from plasmids have shown that, at least for MHV [109], SARS-CoV [110] and IBV [111], the presence of N protein can greatly increase VLP yield. Therefore, while N protein is not necessarily required for envelope formation, N protein plays an important role in forming a complete virion [108].

#### 4.1.4. Genomic mRNA Replication/Genomic RNA Synthesis

N protein binds to both full-length genomic RNA (gRNA) as well as all six sgRNAs but displays an increased affinity for gRNA [112]. The gRNA functions as a template for the viral RNA-dependent RNA polymerase as well as a message for translation [52]. During infection, gRNA is initially transcribed by an early polymerase activity into a genome-sized negative-stranded RNA [113] and then a late polymerase activity transcribes the negative-stranded RNA into a full length gRNA that is bound to polysomes [113] and detected in nucleocapsid structures [114]. Numerous studies have demonstrated that N protein is required for optimal CoV replication [31,115,116,117,118,119,120,121]. The participation of N protein in an early event in RNA synthesis is implied by at least two things: firstly MHV- and SARS-CoV N protein colocalize intracellularly with replicase components at early stages of infection [122,123,124,125]; and secondly, stimulation of gRNA infection is dependent upon N protein translation [52,126]. 

A more clearly defined role for N protein in gRNA synthesis was delineated when an interaction between the SR region of the MHV N protein and a region in the amino terminal segment of the nsp3 subunit of the viral replicase was discovered [51,52]. The N-nsp3 interaction has been specifically mapped to the ubiquitin-like domain (Ubl1) of nsp3, an essential domain to the virus [127]. Moreover, it was also shown that this N-nsp3 interaction is required for N protein to promote optimal infectivity of gRNA [52]. It was proposed that the formation of an initiation complex at the 3' end of the genome requires N protein to tether gRNA to the newly translated replicase via its interaction with nsp3 [52,127]. Considering that the start of negative–strand synthesis is the first step in both genomic replication and transcription [128,129], it is likely that this N-nsp3 interaction is important in both of these RNA-synthetic processes [52]. The role of N protein in MHV gRNA synthesis may not necessarily be limited to early time points of infection as it has also been proposed that N protein sustains ongoing transcription throughout the course of infection [31,130]. The role of CoV N protein in RNA synthesis remains controversial as N protein is not essential for TGEV RNA replication but rather for efficient transcription [31]. 

### 4.2. Cellular Response

#### 4.2.1. Chaperone Activity

RNA chaperone proteins assist the proper folding of nucleic acids [131,132]. Chaperone activity has been postulated as a general activity of all CoV N proteins, and has been demonstrated for TGEV- and SARS-CoV N proteins [30]. It has been proposed that amino acids 117–268 in the central disordered domain (the LKR domain) of the TGEV N protein has chaperone activity [31], and that this region facilitates template switching *in vitro* by decreasing the energy barrier needed to dissociate the nascent minus RNA chain from the gRNA template during discontinuous RNA transcription [30,31]. This facilitation of template switching is required for efficient transcription and may explain how the presence of N protein therefore increases RNA synthesis.

#### 4.2.2. Cell Cycle Regulation

Deregulation of the cell cycle is a common strategy adopted by many RNA (and DNA) viruses aimed at exploiting host cell machinery in order to create a more favorable environment for their survival. One of the MHV nonstructural proteins, p28, induces G_0_/G_1_ cell cycle arrest by inhibiting hyperphosphorylation of retinoblastoma protein (Rb), a step which is necessary for cell cycle progression through late G_1_ into the S phase [133,134,135]. A model was proposed whereby expressed cytoplasmic p28 induces stabilization of p53, and accumulation of p53 causes transcriptional upregulation of p21, a cyclin-dependent kinase (CDK) inhibitor, leading to suppression of cyclin E/CDK 2 activities, and the resulting reduction in G_1_ cyclin-CDK complexes and CDK activities ultimately inhibits Rb hyperphosphorylation [133]. Certain herpes simplex virus gene products [136,137], cytomegalovirus gene products [138,139], and Zta of Epstein-Barr virus [140] have similarly also been shown to arrest cell cycle progression at the G_1_ phase. IBV infection also perturbs cell cycle progression and arrests cells in the S and G_2_/M phases, and this occurs partly through the interaction of IBV nsp3 and DNA polymerase delta [141].

The N protein of SARS-CoV also modulates the host cell cycle by regulating cyclin-CDK activity such that S phase progression is halted. The N protein bears a signature cyclin box-binding region (*RXL* motif), can be phosphorylated by CDK and is therefore a substrate for the cyclin-CDK complex [39]. N protein has been shown to have an inhibitory effect on S phase cyclins CDK4 and to a lesser extent CDK6 [39]. The inhibition of kinases means that Rb remains hypophosphorylated and cannot release E2F1, and the transcription of S phase genes is halted [135]. Similarly to MHV p28, SARS-CoV N protein also inhibits CDK2 activity, meaning that by blocking the activity of both G_1_ and S phase cyclins, N protein doubly ensures the blockage of S phase progression [39]. N protein mediated inhibition of CDK activity; leading to inhibition of Rb phosphorylation, is independent of CDK inhibitors (CDKIs) such as p21, and so it has been suggested that the N protein mimics the role of CDKIs by acting as a competitive inhibitor to CDK4 and CDK2 [39]. The ability of N protein to inhibit CDK4 activity was dependent on the *RXL* motif, whereas its ability to inhibit CDK2 requires at least two mechanisms: the CDK phosphorylation motif and dependence on host cell signaling pathways [39]. It has been postulated that, p28-mediated (MHV) and N protein-mediated (SARS) blocking of the S phase, allows these virus enough time to utilize the cellular raw materials that have been synthesized ahead of the S phase, for replication of its genome, as well as for assembly and budding of progeny particles [142]. These studies provide evidence that even within the CoV family, different proteins can deregulate the cell cycle and that those proteins, in the case of SARS-N protein, can employ multiple mechanisms to achieve this. 

#### 4.2.3. Cell Stress Responses—Host Translational Shutoff

In response to environmental stress, eukaryotic cells reprogram their mRNA metabolism to adapt to stress-induced damage. Translationally stalled mRNAs together with a number of translational initiation factors and RNA-binding proteins are selectively deposited in cytoplasmic stress granules (SGs) [143]. CoVs, like many other viruses, induce host translational shutoff, while maintaining synthesis of their own gene products, as a means to interfere with cellular defense mechanisms [144]. MHV-induces a host translational shutoff that is reminiscent of a cellular stress response; SGs appear, numerous mRNAs including protein translation-related factors are downregulated and levels of phosphorylated translation initiation factors increase [145]. It is important to remember, though that this translational shutoff could also simply be the host cell’s response to the infection.

In SARS-infected HeLa cells that are exposed to arsenite treatment, N protein translocates to cytoplasmic SGs where it colocalizes with poly(A)-binding protein 1 (PARB) and transiently expressed TIA-1 [38], both of which are components of SGs [143]. This localization and pattern was enhanced when the protein was hypophosphorylated (NΔRS), perhaps because NΔRS induced larger ribonucleoprotein (RNP) complex formation [38]. The sequestration of N protein in SGs possibly indicates a role in translational suppression. The nucleocapsid protein of both porcine and respiratory syndrome and rubella virus also interact with PARB, which promotes protein synthesis by circularizing mRNAs, and inhibit host protein synthesis by sequestration of PARB through a stoichiometric mechanism [146,147]. It has been proposed that, at least in the rubella virus, that N-dependent inhibition of translation via PARB binding may facilitate the switch from viral translation to packaging RNA into nucleocapsids [146]. More specifically, binding of the capsid to PARB late in the virus cycle, when the levels of newly synthesized genomes and viral proteins are high, and there is little need for additional replicase components, could prevent recruitment of ribosomes to the nascent 40S RNA. This scenario would favour packaging of the 40S genomic RNA into nucleocapsids [146]. Considering that SARS-N and MHV-N protein has been shown to interact with the PARB hnRNP-A1 [41,148], it is possible that the N-PARB interaction also acts as a switch that redirects viral activity from RNA synthesis to nucleocapsid formation.

The translational suppressive effect of the N protein could also be through its CTD interaction with elongation factor 1α (EF1α), a major translational factor in mammalian cells [75]. N protein of SARS-CoV binds directly to and induces aggregation of EF1α, resulting in inhibition of protein translation [75]. Moreover, EF1α is a multifunctional protein and has unconventional functions related to its association with the cytoskeleton, including interaction with filamentous (F) actin, promotion of F actin bundling and formation of a contractile ring during cytokinesis [149,150,151,152]. N-EF1α therefore blocks EF1α–mediated F actin bundling and the formation of the contractile ring, leading to the formation of multinucleated cells by inhibiting cytokinesis [75]. Multinucleated syncytia of macrophages have been observed in late-phase SARS-CoV but not in other CoV-infected patients [153]. The N protein of hCoV-229E also binds to EF1α, but with significantly lower affinity than SARS-CoV N, and induces multinucleation much more slowly in a substantially smaller fraction of transfected cells [75]. In addition, N-protein mediated inhibition of cytokinesis leads to inhibition of cell proliferation in human peripheral blood lymphocytes (PBLs) [75]. Since actively dividing lymphocytes are a major cell target of SARS-CoV [154], SARS-CoV N may decelerate lymphocyte proliferation and therefore interfere with immune system function [75].

SARS-N protein has also been shown to cause cytoskeletal changes in response to a different type of cell stress, namely serum deprivation [155]. N protein induces actin reorganization in COS-1 monkey kidney cells by down regulating focal adhesion kinase and fibronectin via p38 MAPK pathway activation [155]. 

#### 4.2.4. Viral Pathogenesis—Immune System Interference

N protein plays an important role in viral pathogenesis since anti-N monoclonal antibodies protect mice from lethal infection of JMHV [156]. Coronaviruses seem to have developed mechanisms to overcome the host innate immune response, including the interferon response, a major component of innate immunity. SARS-CoV infected cells do not allow production of interferon and so it seems likely that SARS-CoV, like many other viruses, has developed mechanisms to subvert the interferon response [157]. SARS-CoV N protein is one of three β interferon (IFβ) antagonists, with ORF3b and ORF6 being the other two, and inhibition of IFβ synthesis by N protein is due to inhibition of IRF-3 and NF-kB, two of the transcription factors required for IF gene expression [158]. SARS-CoV N protein inhibits the synthesis of type-1 interferon (1FN) and the CTD of N has been shown to be critical for antagonism of 1FN induction [159]. This inhibition of the interferon response likely contributes to the pathogenesis of SARS-CoV.

#### 4.2.5. Signal Transduction

CoV infection is likely to activate a diversity of host cell signal transduction pathways and kinases, which would lead to phosphorylation of N protein [160]. N protein phosphorylation would therefore only occur at a relatively late stage of the viral cell cycle and may explain why more phosphorylated N protein is incorporated into IBV virions [161].

It has been demonstrated that the SARS-CoV N protein can bind to DNA *in vitro* [55]. Recently, the N protein of hCoV-OC43 was shown to interact with the transcription factor nuclear factor-kappa B (NF-kappaB) [73]. It was reported that hCoV-OC43 N protein potentiates NF-kB activation as a direct result of the ability of the nucleocapsid to bind microRNA miR-9, a negative regulator of NF-kB. It was suggested that this previously undescribed interaction between virus and host is a potential mechanism of immune evasion in RNA viruses because NF-kB required for IF gene expression [73,158]. 

**Table 1:** Summary of the role of coronavirus N protein in the (1) virus life cycle and (2) cellular response.

1. Virus Life Cycle	Function
*1.1 Viral Core Formation*	Primary role of CoV N is packaging the viral genome into long, flexible, helical RNP complexes [77].
*1.2 Viral Assembly*	CoV N protein dimerization [44,54,56] and association with viral genomic RNA [43,79,82,84,85,86] is critical for viral assembly. Interaction amongst the viral structural proteins (N, E, S and M), as well as a host membrane envelope obtained from the site of budding is required for viral assembly [87,88].
*1.3 Virus Budding/envelope formation*	Association of CoV N with the ER-Golgi complex plays a role in virus budding [96,103]. Presence of N results in increased yields of VLPs and complete virion formation [108,109,110,111].
*1.4 Genomic mRNA replication/genomic RNA synthesis*	Intracellular co-localization of N with replicase components is required for RNA synthesis [122,123,124,125]. Translation of N protein is implicated in stimulation of gRNA infection during RNA synthesis [52,126].
**2. Cellular Response**	
*2.1. Chaperone activity*	All CoV N proteins involved in proper folding of nucleic acids by RNA chaperone proteins [131,132].
*2.2. Cell cycle regulation*	SARS-CoV N modulates the host cell cycle by regulating cyclin-CDK activity. Leads to the arrest in progression of S phase [39].
*2.3. Cell stress responses—host translational shutoff*	SARS-N and MHV-N interact with cellular hnRNP-A1 [41,148], which could act as a switch that redirects viral activity from RNA synthesis to nucleocapsid formation. Interaction of N protein CTD with elongation factor 1α (EF1α), a major translational factor in mammalian cells, can suppress translation [75].
*2.4. Viral pathogenesis—Immune system interference*	N protein plays an important role in viral pathogenesis. Mice infected with JMHV protected by anti-N monoclonal antibodies [156]. Synthesis of type-1 interferon (1FN) inhibited by SARS-CoV N [158]. The CTD of N has been shown to be a critical antagonist of 1FN induction [159]
*2.5. Signal transduction*	Activation of host cell signal transduction pathways and kinases leads to phosphorylation of N [160].

NF-kB is also involved in SARS-CoV mediated activation of cyclooxygenase-2 (COX-2), more specifically, N protein binds to NF-kB and CCAAT/enhancer binding protein binding sites on the COX-2 gene promoter [162]. Activation of COX-2, a proinflammatory factor, by SARS-CoV N protein is a likely cause of lung inflammation in SARS-CoV infected patients [162]. SARS-CoV N protein is also known to activate the activator protein 1 (AP1) signal transduction pathway by increasing the amount of transcription factors binding to promoter sequences of c-Fos, ATF2, CREB-1, and FosB [163]. SARS-CoV N protein induces apoptosis of COS-1 monkey kidney cells in the absence of growth factor by down-regulating extracellular-signal-regulated kinase (ERK) and up-regulating c-Jun N-terminal kinase (JNK) and p38 mitogen-activated protein kinase (MAPK) pathways, with activation of p38 MAPK also causing actin reorganization in serum-deprived cells [155].

## 5. Conclusions

The coronavirus N protein is abundantly produced within infected cells. N has multiple functions, including binding to viral RNA to form the ribonucleocapsid and has also been proposed to have roles in virus replication, transcription and translation. In host cells, N proteins have been shown to cause deregulation of the cell-cycle [70,142], inhibit the production of interferon [158], up-regulate the production of COX2 [162], up-regulate the activity of AP1 [163], and induce apoptosis in serum deprived cells [155]—of all which may have possible pathological consequences [164]. Several excellent reviews on the coronavirus N protein have previously been published [165,166], including one on the structure and function of the SARS-CoV N and its interaction with nucleic acids [19]. Notwithstanding these reviews, the manner in which coronavirus N proteins carry out its roles during the viral life cycle is still not clearly understood. An important piece of missing information lies in the difficulty in resolving the atomic structure of the RNP complex, which has been hampered by low solubility of the RNP complex and the labile nature of the full-length N protein. In addition, in order to determine whether the RNPs from various coronaviruses share a common structural code, the structure of different coronavirus RNPs need to be resolved [19]. 

Direct intraviral protein-N interactions identified to date include the interaction between N and M [61,92,93] and N and nsp3a, a component of the viral replicase [10,52]. Additionally, in MHV-infected cells, monoclonal anti-N antibody co-immunoprecipitates both M and S proteins; this N-S interaction is not a direct one though. Rather, it is due to the interaction between S and M protein [93], where the S protein forms complexes with M protein in the endoplasmic reticulum (ER) [167,168]. The identification of host proteins targeted by viral proteins during the infection process provides important insights into mechanisms of viral protein function [169]. To date, the interaction of N with numerous host cell proteins have been identified, including hCypA [170], proteasome subunit p42 [171], the B23 phosphoprotein [172,173], Smad3 [174], nRNP-A1 [148], the chemokine CXCL16 [175], translation elongation factor-1 alpha [75], cellular pyruvate kinase protein [176], 14-3-3 [39] and nucleolin [73,177]. More recently, a study using high-throughput mass spectrometry identified a list of cellular proteins that could potentially interact with the IBV N protein [177]. Comparative studies between various coronavirus N protein interactions could provide valuable information on host specificity and evolution of the interactions between N and host cell proteins. In turn, this may offer insight into the development of novel antiviral therapeutics that target interactions between host cell proteins and the N protein [177,178].

SARS-CoV N protein is extremely antigenic. SARS-CoV infection causes a highly restricted, immunoglobulin G-dominated antibody response that is directed most frequently and predominantly at the nucleocapsid [179]. DNA vaccines encoding SARS-CoV N protein generate a strong N-specific humoral and T-cell-mediated response and significantly reduce the viral titre of challenging vaccinia virus in C57BL/6-vaccinated mice [180]. Importantly though, another study suggests that N does not induce virus neutralizing antibody and as a result, provides no protection to infection in hamsters [181]. In the diagnosis/screening hCoV-OC43, rabbit poloyclonal antibodies demonstrated greater immunoreactivity to the central (LKR) region and CTD than the NTD of N protein in serum samples, highlighting that LKR region is a strong candidate for use in the design of diagnostic tools [182]. Understanding the role of N in coronavirus infection could lead to the development of novel therapeutics that could potentially be used to combat the threat posed by the emerging lethal human coronaviruses identified in recent times.
